# Supplementary material for: Human interictal epileptiform discharges are bidirectional traveling waves echoing ictal discharges
Source: eLife. 2022 Jan 20;11:e73541. doi: 10.7554/eLife.73541 (PMC8813051; doi:10.7554/eLife.73541)
Supplement: Transparent reporting form [file elife-73541-transrepform1.pdf]

Westbrook Centre
Milton Road
Cambridge CB4 1YG, UK

P 01223 855340
W elifesciences.org
T @elife

eLife's transparent reporting form

We encourage authors to provide detailed information *within their submission* to facilitate the interpretation and replication of experiments. Authors can upload supporting documentation to indicate the use of appropriate reporting guidelines for health-related research (see EQUATOR Network), life science research (see the BioSharing Information Resource), or the ARRIVE guidelines for reporting work involving animal research. Where applicable, authors should refer to any relevant reporting standards documents in this form.

If you have any questions, please consult our Journal Policies and/or contact us: editorial@elifesciences.org.

Sample-size estimation

- You should state whether an appropriate sample size was computed when the study was being designed
- You should state the statistical method of sample size computation and any required assumptions
- If no explicit power analysis was used, you should describe how you decided what sample (replicate) size (number) to use

Please outline where this information can be found within the submission (e.g., sections or figure legends), or explain why this information doesn't apply to your submission:

Statistical hypothesis testing in this manuscript was largely performed across large numbers of detected interictal epileptiform discharges (IED). Post hoc power analysis with G*power were carried out, in order, for:

1) One test was carried out across subjects; a McNemar Test showing significantly more IEDs defined from MUA in the seizure core vs. penumbra had an effect size of w = sqrt(CHI^2/n) = sqrt(2957/10) = 17.2, yielding power equal to 1.
2) Median tests of discharge angle difference from the direction of the ictal wavefront. One-tailed test with mean difference of 2.59 radians and the smallest sample size of traveling waves (n = 1429) in the recruited group, for which this test is relevant, yields power of 1.
3) Mann-Whitney U tests for sub-distribution speed differences have sufficient sample sizes to have power equal to one with two-tailed tests.
4) A two-sample proportion test for IED sub-distribution proportions with a minimum effect size of w = sqrt(CHI^2/n) = sqrt(347.6/1429) = 0.5, yielding power equal to 1.

Based on a priori power analyses consisting of bootstrap simulations (probability that the permutation test statistic is greater than the test critical value), all permutation tests were adequately powered given the large numbers of microelectrodes (for traveling wave permutation tests) and discharges (for tests of nonuniformity, differences from ictal wavefront, Kuiper tests for goodness-of-fit, and neurophysiological features of IEDs).

Replicates

eLife Sciences Publications, Ltd is a limited liability non-profit non-stock corporation incorporated in the State of Delaware, USA, with company number 5030732, and is registered in the UK with company number FC030576 and branch number BR015634 at the address Westbrook Centre, Milton Road, Cambridge CB4 1YG.

- You should report how often each experiment was performed
- You should include a definition of biological versus technical replication
- The data obtained should be provided and sufficient information should be provided to indicate the number of independent biological and/or technical replicates
- If you encountered any outliers, you should describe how these were handled
- Criteria for exclusion/inclusion of data should be clearly stated
- High-throughput sequence data should be uploaded before submission, with a private link for reviewers provided (these are available from both GEO and ArrayExpress)

Please outline where this information can be found within the submission (e.g., sections or figure legends), or explain why this information doesn't apply to your submission:

Biological replication was carried out across patients (n = 10 total, n = 6 recruited, and n = 5 bimodal and recruited) and seizures (N = 10 recruited seizures, and n = 5 penumbral seizures). Experiments across IEDs were carried out in each patient.

IED amplitude outliers (> 2* IQR) were rejected in order to ensure the dataset did not include large, artifactual electrophysiological transients.

eLife Sciences Publications, Ltd is a limited liability non-profit non-stock corporation incorporated in the State of Delaware, USA, with company number 5030732, and is registered in the UK with company number FC030576 and branch number BR015634 at the address Westbrook Centre, Milton Road, Cambridge CB4 1YG.

Statistical reporting

- Statistical analysis methods should be described and justified
- Raw data should be presented in figures whenever informative to do so (typically when N per group is less than 10)
- For each experiment, you should identify the statistical tests used, exact values of N, definitions of center, methods of multiple test correction, and dispersion and precision measures (e.g., mean, median, SD, SEM, confidence intervals; and, for the major substantive results, a measure of effect size (e.g., Pearson's r, Cohen's d)
- Report exact p-values wherever possible alongside the summary statistics and 95% confidence intervals. These should be reported for all key questions and not only when the p-value is less than 0.05.

Please outline where this information can be found within the submission (e.g., sections or figure legends), or explain why this information doesn't apply to your submission:

Statistics are reported clearly in the results section of the text, including test statistics and degrees of freedom, typically before the relative figure reference. Statistical methodology for each section is described in the methods. Exact p-values are reported, except in cases when a result corresponds to multiple p-values, in which case the minimum p-value is reported. Raw data are shown in all figures, except for IED direction distributions, which are more clearly viewed as polar histograms.

(For large datasets, or papers with a very large number of statistical tests, you may upload a single table file with tests, Ns, etc., with reference to sections in the manuscript.)

Group allocation

- Indicate how samples were allocated into experimental groups (in the case of clinical studies, please specify allocation to treatment method); if randomization was used, please also state if restricted randomization was applied
- Indicate if masking was used during group allocation, data collection and/or data analysis

Please outline where this information can be found within the submission (e.g., sections or figure legends), or explain why this information doesn't apply to your submission:

We examined a group of 10 epilepsy patients who were implanted with microelectrode arrays. These recordings are rare, and this manuscript constitutes the largest sample of patients published in a manuscript to date. Subgroups of these patients were examined and compared based on the features of their microelectrode array recordings, which are clearly described in the introduction, results, and methods. Two variables were used to subgroup patients:

1) Whether their arrays exhibited the characteristic tonic firing of the ictal wavefront that signals an array's recruitment into seizing brain tissue ('recruited' group), or not ('penumbral' group).
2) Whether patients had bimodal IED distributions or not, based on clustering and nonparametric goodness-of-fit tests.

eLife Sciences Publications, Ltd is a limited liability non-profit non-stock corporation incorporated in the State of Delaware, USA, with company number 5030732, and is registered in the UK with company number FC030576 and branch number BR015634 at the address Westbrook Centre, Milton Road, Cambridge CB4 1YG.

Additional data files ("source data")

- We encourage you to upload relevant additional data files, such as numerical data that are represented as a graph in a figure, or as a summary table
- Where provided, these should be in the most useful format, and they can be uploaded as "Source data" files linked to a main figure or table
- Include model definition files including the full list of parameters used
- Include code used for data analysis (e.g., R, MatLab)
- Avoid stating that data files are "available upon request"

Please indicate the figures or tables for which source data files have been provided:

We have provided the full postprocessed dataset on OSF at: https://osf.io/zhk24/, and analysis code at: https://github.com/elliothsmith/IEDs.

eLife Sciences Publications, Ltd is a limited liability non-profit non-stock corporation incorporated in the State of Delaware, USA, with company number 5030732, and is registered in the UK with company number FC030576 and branch number BR015634 at the address Westbrook Centre, Milton Road, Cambridge CB4 1YG.
